# The British Pharmacopœia

**Published:** 1914-10-03

**Authors:** A. J. Cockington

**Affiliations:** Acting Registrar. General Medical Council, 299 Oxford Street, W.


### THE EDITOR'S LETTER-BOX.

### The British Pharmacopœia.

*To the Editor of* The Hospital.

Sir,—At its meeting on the 15th instant the Executive Committee of this Council received a report by the Registrar on the postponement of publication of the British Pharmacopœia, 1914, which had been rendered necessary by the events of the past month.

It was resolved by the Committee, on behalf of the Council, that copies, in advance of publication, should be made accessible to the public for inspection at the offices of the Council in London, Edinburgh, and Dublin, on Thursday, October 1, 1914, at 10 a.m., and thereafter from 10 a.m. to 4 p.m. daily; and that a copy for review should on the same day be placed in the hands of the editor of each of the medical and pharmaceutical journals in the Registrar's list.

The official publication of the work will be made by notices in the *Gazettes* on Thursday, December 31, 1914, on which day copies will be on sale at the price of 10s. 6d. net, by the publishers, Messrs. Constable and Co., Ltd., 10 Orange Street, Leicester Square, London, W.C.—Yours faithfully,

A. J. Cockington, Acting Registrar.

General Medical Council, 299 Oxford Street, W.
September 22, 1914.

